# The impact of the COVID-19 pandemic on paramedics’ mental health in Greece

**DOI:** 10.1192/j.eurpsy.2021.736

**Published:** 2021-08-13

**Authors:** L.E. Peppou, T. Giannouchos, M. Economou, A. Paschali

**Affiliations:** 1 First Department Of Psychiatry, National & Kapodistrian University of Athens, Athens, Greece; 2 Unit Of Social Psychiatry & Psychosocial Care, University Mental Health, Neurosciences and Precision Medicine Research Institute “Costas Stefanis” (UMHRI), Athens, Greece; 3 Pharmacotherapy Outcomes Research Center, College Of Pharmacy, University of Utah, Utah, United States of America; 4 Faculty Of Nursing, Department Of Mental Health & Behavioral Sciences, National & Kapodistrian University of Athens, Athens, Greece

**Keywords:** coronavirus, frontline workers, common mental disorders, coping skills

## Abstract

**Introduction:**

Converging evidence substantiates a negative impact of the COVID-19 pandemic on the mental health of frontline workers. Nonetheless, there is paucity of research on paramedics.

**Objectives:**

To estimate the prevalence of stress, anxiety and depression in frontline paramedics in the Athens region, Greece, and to investigate the coping skills that are associated with less favourable mental health outcomes

**Methods:**

A total of 100 ambulance paramedics participated in the study. The online questionnaire encompassed the DASS-21 for assessing mental health outcomes and the Brief-COPE for measuring coping skills. Information about socio-demographic characteristics and personal/relatives’ vulnerability to COVID-19 was also gleaned.

**Results:**

The prevalence for moderate to severe cases was found to be 7.2% for stress, 9.4% for anxiety and 11.3% for depression. Multiple linear regression analysis indicated that men demonstrated significantly higher stress [B = -2.28, 95%CI = -3.88 - -0.68] and depression compared to women [B = -1.69, 95%CI = -3.19 - -0.19]. Similarly, the use of denial was found to be associated with higher stress [B = 0.69, 95%CI = 0.11 -1.37] and anxiety [B= 0.55, 95%CI = 0.13 – 0.98]. Moreover, emotional support was linked to heightened anxiety [B= 0.71, 95%CI = 0.36 – 1.06] and self-distraction to depression [B = 0.60, 95%CI = 0.16 – 1.04]. Personal or relatives’ vulnerability to COVID-19 did not impinge on mental health outcomes.

**Conclusions:**

Healthcare initiatives should be tailored at the mental health needs of frontline paramedics, especially men. Psychosocial interventions should target maladaptive coping, especially the use of denial.

